# Sustainable Ecosystem Services of a Time-Honored Artificial River Ecosystem—Enlightenments from the Carp Brook, in Northern Fujian Province, China

**DOI:** 10.3390/ijerph20053959

**Published:** 2023-02-23

**Authors:** Yuliang Li, Ran Yi, Lin Liu, Feng Chen

**Affiliations:** 1 Third Institute of Oceanography, Ministry of Natural Resources, Xiamen 361005, China; 2 Institute of Urban Environment, Chinese Academy of Sciences, Xiamen 361021, China; 3 School of Physics, Huazhong University of Science and Technology, Wuhan 430074, China; 4 College of Computer and Information Engineering, Xiamen University of Technology, Xiamen 361024, China; 5 Big Data Institute of Digital Natural Disaster Monitoring in Fujian, Xiamen University of Technology, Xiamen 361024, China

**Keywords:** river ecosystem, social-ecological systems, ecosystem services, folk customs, artificial ecosystem, the Carp Brook

## Abstract

Building a harmonious relationship between human society and river ecosystems has attracted much attention from both government officials and the academy community. Based on the perspective of social-ecological systems (SES), taking the Carp Brook (located in northern Fujian Province, China) as an example, the construction and maintenance of a time-honored artificial river ecosystem was investigated, and its ecosystem services were analyzed. Findings show that the Carp Brook was constructed through a series of ecological engineering, including a transformation of the river channel, building a stable habitat, and breeding carps. The carps have been protected effectively by some folk customs, such as village regulations and folk belief. Meanwhile, the water quality has been maintained through some engineering and institutional measures, which were completed by the local government and villagers. Furthermore, some cultural elements with local characteristics have been formed during the long years of coexistence between human society and the Carp Brook. Based on a healthy ecosystem and abundant culture elements, the Carp Brook provided continuous ecosystem services to human society for more than 800 years, including regulation services (e.g., water purification and flood control) and cultural services (e.g., tourism, research and education, inspiration). Major enlightenments from the Carp Brook are: (a) the Chinese traditional view of nature is important for the construction and maintenance of an artificial ecosystem; (b) traditional folk customs have a strong binding force regarding the protection of the ecosystem; and (c) the choice between material and immaterial services should be made carefully.

## 1. Introduction

Ecosystem services are the benefits people obtain from ecosystems, which can be defined as four broad categories, including provisioning services (e.g., timber, fish, and freshwater), regulating services (e.g., climate regulation and pest control), cultural services (e.g., tourism and spiritual benefits), and supporting services (e.g., soil formation and the nutrient cycle) [1]. In particular, as an important channel of material and energy circulation in the natural world, rivers provide multitudinous ecosystem services to human beings. However, with rapid social and economic development, improper utilization of the river ecosystem including excessive pollution discharge, disordered mining, and overfishing has caused a series of irreversible ecological damages. These damages have resulted in serious influence on the supply of river ecosystem services [2,3]. Humans must find a balance between the utilization and protection of river ecosystems and realize the sustainable supply of river ecosystem services. Nowadays, some studies on river ecosystem services provide the foundation with sustainable river development and management. Most of these studies have conducted qualitative and quantitative analyses of river ecosystem services. For example, Thiele et al. [4] applied an indicator framework for spatially assessing the offered cultural ecosystem services in German river landscapes. Zhang et al. [5] used the SolVES model to evaluate the social value of ecosystem services on the east bank of the Fenghe River and found the contribution of different environmental variables to social value. Karabulut et al. [6] mapped and assessed water provisioning services and associated benefits to support the ecosystem–water–food–energy nexus. In addition, some studies have focused on the relationship between human well-being and river ecosystem services [7,8,9] and the drivers of changes in river ecosystem services [10,11,12]. However, these studies almost focused on natural river ecosystems, and there has been a lack of research on artificial river ecosystem. Theoretical foundation for the construction and development of artificial river ecosystems and the improvement of the supply of river ecosystem services need to be further strengthened.

The concept of social-ecological systems (SES) acknowledges the interaction of human society and ecosystems through the flow of materials, energy, and information between two systems [13,14]. The SES is useful to increase recognition of the reciprocal feedback mechanism between human society and the ecosystem [15], and to facilitate obtaining experience for the sustainable supply of artificial river ecosystem services [16,17]. Based on the perspective of SES, this study took a time-honored artificial river ecosystem—the Carp Brook (located in the Fujian Province, China)—as an example to identify the influence of human society on the Carp Brook from multiple dimensions, and to explore its feedback to human society using ecosystem services as indicator. This study aimed to obtain enlightenment on the lasting existence and sustainable ecosystem services of the Carp Brook. The major findings are mainly based on investigations on the construction and maintenance of the Carp Brook and on the analysis of its ecosystem services. The immaterial services of river ecosystems can also bring continuous and considerable well-being to human beings. Both the traditional view of nature and folk customs are still important in rural environment protection. These enlightenments from the Carp Brook may provide theoretical support for river ecosystems and even rural ecosystem management.

## 2. Materials and Methods

### 2.1. Study Area

**Figure 1:**
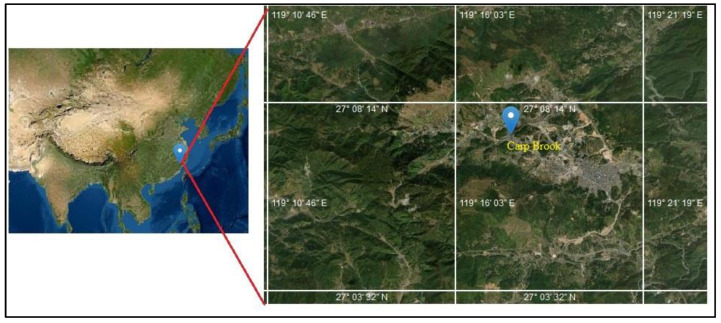
Location of the Carp Brook within Puyuan village, Zhouning county, in Southeast China’s Fujian province. The base map was from the ESRI world imagery.

The Carp Brook is a branch of Dongyang Stream in Zhouning County, Fujian Province, China (Figure 1). The midstream of the Carp Brook is about 3 m wide and 600 m long, passing through the Puyuan village, where about 7000 to 8000 colorful carps live. Therefore, it is named “the Carp Brook”. The Carp Brook has been famous at home and abroad due to its unique natural and cultural landscape and has been listed in the World’s Guinness Records for the world’s only carp grave, carp burial, carp sacrifice, and the oldest in history. The folk customs which originated along the Carp Brook for carp protection were listed as the Intangible Cultural Heritage of Fujian Province [18].

### 2.2. Methods and Data Source

#### 2.2.1. Methods

Based on SES and the ecosystem services theory, this study analyzed the interaction between human society and an artificial river ecosystem through qualitative and quantitative methods. It aimed to gain experience for ecosystem management. The main investigations were conducted from August to October 2022, through literature research method and field investigation method. 

Literature research method included collection and analysis of papers, books, statistic data, and news reports related to the study area. Field investigation method included field trips and interviews. In addition to the literature research, we investigated the upstream, midstream, and downstream of the Carp Brook, and recorded the ecological engineering facilities and cultural elements along it. We interviewed four staff members of the Puyuan Town Government to learn about the development and conservation of the Carp Brook. Meanwhile, we interviewed four staff members of the Scenic Area Administration Committee of the Carp Brook to learn about the development and management of tourism resources. To understand the history of the village, we interviewed two staff members of the Puyuan Village Heritage Management Association. Six Puyuan villagers were interviewed to discuss their living experience, changes in well-being, and participation in the management of the Carp Brook, while six tourists were randomly interviewed to investigate their experience of visiting the Carp Brook.

#### 2.2.2. Data Source

The data for qualitative analyses were collected from the Local Records of Zhouning County (1992), the Zhouning Yearbook (2015–2021), research papers and books, interview records, photographs in the field, websites, and some materials provided by the local offices.

In addition, several sensory indicators, microbiological indicators and chemical indicators were used to indicate the water quality of the Carp Brook, which were provided by the Environment Protection Office of Puyuan Town Government. Meanwhile, the annual number of visitors was used to indicate the tourism service, which was collected from the Scenic Area Administration Committee of the Carp Brook. The number of relevant research papers was used to indicate the research and education service, which was estimated through the China National Knowledge Infrastructure literature platform (https://www.cnki.net/, assessed on 30 October 2022).

#### 2.2.3. Data Analysis

The descriptive date analysis method was used in this study. It included extracting information from the Local Records of Zhouning County (1992), the Zhouning Yearbook (2015–2021), research papers and books, interview records, photographs in the field, websites, and some materials provided by the local offices for descriptions of the construction, maintenance, and ecosystem supply of the Carp Brook.

## 3. Results

### 3.1. Construction and Maintenance of the Carp Brook

#### 3.1.1. Construction of the Carp Brook

The Zheng (a family name of Chinese) people had migrated to Dongyang Stream basin in 1209 A.D.; they then built the Carp Brook ecosystem by transforming the river channel of the branch of Dongyang Stream and breeding carps in it. The purposes of the Zheng people to breed carps in the river were: (a) to use carps as indicator organisms to test the river water for poison; (b) to enhance the self-purification capacity of the river ecosystem; (c) to improve the river landscape [19]. 

**Figure 2:**
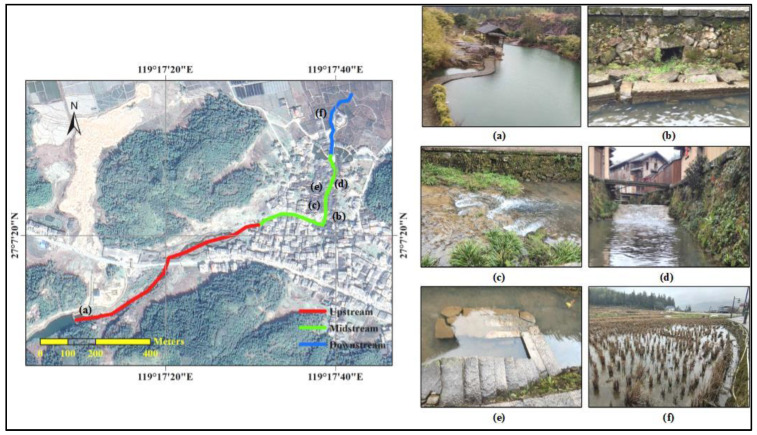
Ecological engineering facilities along the Carp Brook. The sketched map of the Carp Brook is indicated in bold lines: the upstream (red), the midstream (green), and the downstream (blue). Typical facilities are: (**a**) dam, (**b**) bank with caves, (**c**) weeds, (**d**) dam, (**e**) L-shaped sewer, and (**f**) farmland.

The Zheng people divided the branch of Dongyang Stream into three sections (upstream, midstream, and downstream) when the Carp Brook ecosystem was constructed, with each section taking different ecological engineering measures (Figure 2).

(1) Upstream

A dam was built upstream of the branch of Dongyang Stream to divide its water flow into two parts. Some of the water flows into the pools and ditches in Puyuan village, which are located along the midstream of the branch of Dongyang Stream. Thus, domestic water for Puyuan villagers can be provided by these pools and ditches, while the domestic sewage can be taken away by the ditches. Other parts of the water flows into the midstream of the branch of Dongyang Stream through diversion channels. There are some sluices set on the dams at the entrance of diversion channels which can adjust the water source and volume.

(2) Midstream

The midstream was transformed into an “S” shape, on which dams were constructed for breeding the carps. The S-shaped channel and the dams constructed on it can slow down the flow of the midstream, providing a stable habitat for the carps. Banks of the midstream were built with irregular granite and cobblestone, and some caves were left for carps to hide and roost, preventing the carps from being washed away during flash floods. Similarly, some L-shaped sewers were built on the riverbed for carps to hide in the flood. Moreover, some weeds were planted along the banks bordering the water. These weeds not only enriched the landscape of the river ecosystem, but also served as a hiding place to avoid the carps being washed away in the flood.

(3) Downstream

The downstream flows into a large area of farmland northeast of Puyuan Village for irrigation. The domestic sewage brought by the ditches in Puyuan village to the downstream contains some nutrients, making its irrigation effect better than the general river water.

#### 3.1.2. Protection of the Carps

(1) Protect the carps through village regulations.

Village regulations are traditional self-management rules with characteristics of autonomy, self-discipline, contractual, regional, and mandatory, which are made and complied in a certain Chinese rural territorial scope by certain organizations or groups [20,21]. In the early Ming Dynasty, the Zheng people in Puyuan village began to make village regulations for carp protection. The regulations for carp protection have been inherited successively, with the Puyuan villagers forming a collective mind. In particular, fishing carps is prohibited; a person who catches carp from the Carp Brook will be punished. Under the constraints of carp protection regulations, a Puyuan villager will never eat carps, even in period of local famine, and outsiders are not allowed to catch carps from the Carp Brook. Moreover, any caught carps must be released. If Puyuan villagers encounter a caught carp outside Puyuan village, they must buy and release it into the Carp Brook. The binding force of the regulations is still very strong today. For example, in 2021, the Board of Zheng Ancestral Hall punished a Puyuan villager who caught carps from the river.

(2) Protect the carps through folk belief.

Since the Ming Dynasty, the patriarchs of the Zheng people have made up fairy tales to convince the villagers that the carps were the incarnation of the gods and carp fishing would lead to a disastrous disease [19,22]. In an ancient rural society with low productivity, the prestigious people that fabricated and told these fairy tales gave carps sacred character and formed folk beliefs about carps through generations. Currently, the folk beliefs not only play an important role for the protection of carps, but have also become unique cultural elements.

(3) Other measures for carp protection.

Except village regulations and folk beliefs for carp protection, Puyuan villagers organized the Carp Protection Team and invented martial arts for carp protection spontaneously. The martial arts for carp protection has been identified as the Intangible Cultural Heritage of Zhouning County.

#### 3.1.3. Maintenance of the Water Quality

The local government and Puyuan villagers have taken a series of engineering measures and institutional measures to keep the Carps Brook clean. The local government mainly completed engineering measures, including the construction of a sewage pipe network and sewage treatment plant, the diversion of rain and sewage, river diversion, and river dredging [23,24,25,26,27,28,29]. The institutional measures were made by Puyuan villagers spontaneously, including: (a) the River Chief System, whereby a River Chief is responsible for the water quality of the Carp Brook; (b) the Civilized Behavior Guide Team, which is composed of Puyuan villagers and prevents the pollution of the river by patrolling; and (c) the Sewage Discharge Supervisor, whereby some Puyuan villagers supervise the daily sewage discharge of households in their village.

### 3.2. Preservation of Cultural Elements

At present, the most important cultural elements of the Carp Brook ecosystem are the folk customs about the carp and the folk belief places along the river. The folk customs include the village regulations and folk beliefs for carp protection, the carp sacrifice and burial ceremony, etc. The village regulations and the folk beliefs for carp protection have been described above. This section mainly introduces the carp sacrifice and burial ceremony, and the folk belief places along the Carp Brook.

#### 3.2.1. The Carp Burial and Sacrifice Ceremony

The carp burial and sacrifice ceremony are the distinctive folk customs in Puyuan village. If a carp dies in the Carp Brook, the villagers will hold a carp burial and sacrifice ceremony. The fish burial ceremony is led by an old man, holding a copper pan on which the carp to be buried. Behind him are Puyuan villagers attending the funeral. People read the funeral oration, light firecrackers, bang drums and gongs, and burn joss sticks at the funeral, just like burying a deceased relative or friend.

#### 3.2.2. The Folk Belief Place along the Carp Brook

**Figure 3:**
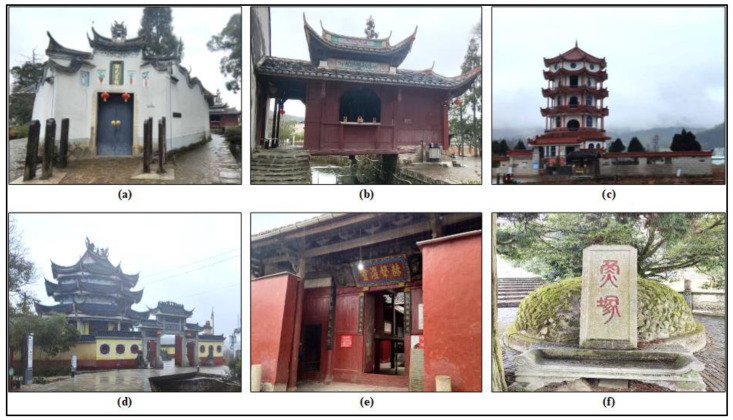
Several folk belief places along the Carp Brook: (**a**) Zheng’s Ancestral Hall, (**b**) Guanyin Gallery, (**c**) Wenchang Tower, (**d**) Guanyin Tower, (**e**) Tianhou Temple, and (**f**) Carp Grave.

The folk belief reflects the world and life view of rural residents [30]. As the space carrier of folk belief, folk belief places contain rich human emotion, collective memory, and cultural spirit, so they have distinct local characteristics [31]. The folk beliefs in Puyuan village can be divided into two major systems: ancestor worship and belief in God. The two systems have their corresponding public belief places along the Carp Brook (Figure 3). Specifically, the folk belief place related to ancestor worship is Zheng’s Ancestral Hall, whereas the belief places related to God belief are the carp grave and temples for various gods [32].

### 3.3. Ecosystem Services of the Carp Brook

#### 3.3.1. Regulation Services

Regulation services of the Carp Brook include water purification and flood control. There are various ecological processes of the Carp Brook ecosystem accompanied by material and energy cycles, through which water purification service are provided. The main pollutants discharged into the Carp Brook are sewage and food scraps from households along the river. On the one hand, pollutants in sewage can be assimilated by primary producers (such as algae) in the river, while a large number of carps as predators of primary producers can effectively control their population size and prevent algal bloom. On the other hand, the carps directly consume the food scraps and purify the water. Consequently, the water quality indicators of the Carp Brook meet the grade I standard for the source quality of drinking water (Table 1). In addition, the water storage and drainage functions of the Carp Brook have played an irreplaceable role for relieving the damage of floods throughout the history of Puyuan village [18].

#### 3.3.2. Cultural Services

(1) Tourism service

Because they have lived with humans for a long time, the carps in the Carp Brook are not afraid of humans. Visitors can appreciate the colorful carps or throw bait into the water, experiencing the fun of interacting with feeding carp. In particular, during their migration to upstream for spawn, the carps can be seen crossing the dam in the river, as in the Chinese legend of “Carp jumps over the Dragon Gate”. In addition, the folk customs and the folk belief places along the Carp Brook are worth visiting and exploring. 

The local government set up a tourism company to develop the tourism resource of the Carp Brook ecosystem together with social capital [25,27,33]. The tourism company is a daily manager of the Carp Brook ecosystem. It has improved tourism facilities, while also keeping the river clean and building museums to demonstrate the cultural elements of the Carp Brook [23,24,25,26,27,28,29]. The local government has increased the popularity of the Carp Brook through many ways, such as making tourism promotional films, holding tourism cultural festivals, participating in tourism fairs at home and abroad, and inviting domestic and foreign travel agencies and professionals to make field visits to Carp Brook [23,24,25,26,27,28,29]. In particular, Nicholas, the chairman of the World Geopark Network Association, visited the Carp Brook in 2017 and gave high praise to its natural and cultural landscape [26].

(2) Research and education

At the China National Knowledge Infrastructure literature platform, with “Zhou Ning” and “the Carp Brook” as the themes, 17 reference papers were found in total, including journal papers, dissertations, and conference papers (up to 30 October 2022). Their themes involve tourism, geography, literature, architectural science, macroeconomics, and sustainable development. A book titled “Culture Research of the Carp Brook” was published. The village regulations for carp protection in Puyuan village were recorded in the book titled “The Village Regulations and Rules of Fujian Province”. Moreover, the essay “Wonderful Carp Brook” has been included as a teaching material in Chinese textbooks of Beijing elementary schools.

(3) Inspiration

There are some films and television works inspired by the Carp Brook, including two films named “Old Shadow” and “Zhou Ning’s Dream”, respectively. Meanwhile, a documentary named “The Wonderful Fate between Carps and Human” and a feature program in the columns called “Remember Your Homesickness” were made by China Central Television.

## 4. Discussion

### 4.1. The Interaction between Puyuan Village Society and the Carp Brook Ecosystem

**Figure 4:**
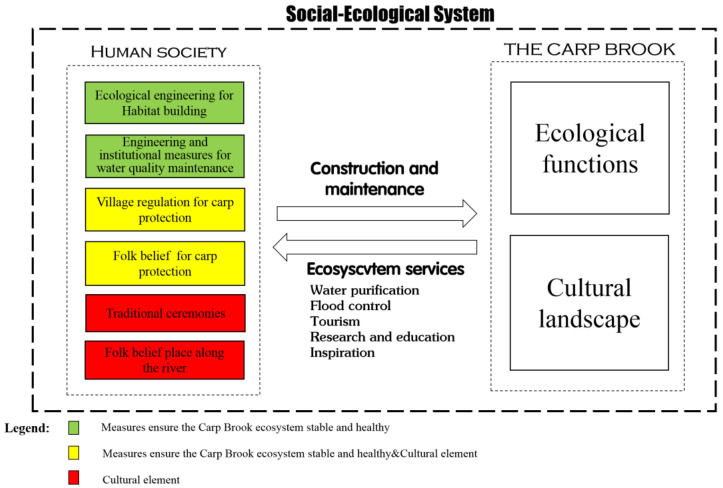
The interaction between the Puyuan village society and the Carp Brook ecosystem.

For more than 800 years, Puyuan villagers (currently about 5000 people, most of whom are the Zheng people) have taken various measures to keep the Carp Brook ecosystem healthy and to ensure its sustainable ecosystem service supply. The Carp Brook ecosystem was constructed through a series of ecological engineering (Figure 2); its water quality is maintained through some engineering and institutional measures, and its dominant species (the carps) are protected through folk customs such as village regulations and folk beliefs. Meanwhile, during the long years of coexistence between humans and the Carp Brook ecosystem, some local characteristic cultural elements have been formed, such as village regulations, folk beliefs, traditional ceremonies, and folk belief places. All of them ensure that the ecosystem services of the Carp Brook ecosystem is sustainable, including water purification, flood control, tourism, research and education, and inspiration (Figure 4). These demonstrate a long-term virtuous cycle between a rural society and an artificial river ecosystem. In other words, as a SES, the Carp Brook ecosystem is sustainable.

### 4.2. Some Characteristics of the Carp Brook

#### 4.2.1. The Carp Brook Is a Time-Honored Artificial River Ecosystem

There are also some successful cases of artificial river ecosystems which have significantly improved human well-being. The Cheonggyecheon River in Downtown Seoul is a typical case [34]. Since the 15th century, the Cheonggyecheon River had been degraded due to human activities. In the 1970s, the Cheonggyecheon River was covered by a busy, multi-lane roadway, and an elevated highway. Because of serious traffic congestion and air pollution, a two-year project costing nearly USD 1 billion began in 2003, and the river has since been re-exposed. The restored Cheonggyecheon River has brought some ecological benefits, such as an improvement in air quality, mitigation of the heat-island effect, and an increase in biodiversity, as well as some socio-economic benefits, manifested as an increase in tourists, property values, and commercial activities along the river. Nowadays, to keep the river flowing, water must be pumped from the Han River and underground water reserves at a cost of more than JPY 200 million (approximately USD 1.52 million) a year. As artificial rivers, both Carp Brook and Cheonggyecheon River have brought obvious benefits to human society. However, the Cheonggyecheon River had been seriously degraded by the destruction of human activities, until this disappeared in the 1970s. Currently, the Cheonggyecheon River is a restoration project that requires significant costs to complete and maintain. In contrast, the Zheng people in the ancient farming society upholding the Chinese traditional view of nature had reasonable goals and used scientific engineering measures to construct the Carp Brook, which has coexisted in harmony with human society for hundreds of years.

#### 4.2.2. The Carp Culture Are Attractive

Similar to the Carp Brook, fish in some other rivers also have cultural importance, such as giant catfish in Chiang Khong [35]. The main activities surrounding the giant catfish include the giant catfish ritual, the giant catfish museum, and the Thai Department of Fisheries’ giant catfish breeding program, which attracts a large number of visitors to Chiang Khong. The culture of the giant catfish in Chiang Khong can also be developed as a tourism resource to improve the livelihoods of local people; however, it does not play a significant role in the protection of the river ecosystem. Since the Ming Dynasty, Puyuan villagers have formed the local collective consciousness of carp protection through folk customs such as village regulations and folk beliefs. Whether in a famine or in modern society, the folk customs for carp protection can effectively protect the river ecosystem. In addition, other studies have also demonstrated the importance of Chinese folk customs in water resources management [36], soil erosion prevention [37], forest protection [38], and wildlife protection [39].

#### 4.2.3. The Carp Brook Is a Co-Managed Fish Conservation Zone

Local government plays a leading role in the management of the Carp Brook ecosystem. It spends a lot of financial funds to construct environmental protection facilities and takes engineering measures to keep the river ecosystem healthy. Furthermore, it set up a tourism company to develop the tourism resource of the Carp Brook ecosystem. The tourism company improves tourism facilities and maintains the environment, from which the economic value of tourism service is realized with its operation. The villagers in Puyuan are not only the inheritors of cultural elements but are also the participants in protecting the Carp Brook ecosystem with their inherited collective mind. In a word, the government, tourism company, and villagers have clear responsibilities and complementary functions in the management of the Carp Brook ecosystem. Similarly, many countries in Southeast Asia have adopted community-based management or co-managed Freshwater Protected Areas for fish conservation in river and lake environments [40]. For instance, the Restricted Area for Fishing at Tha Song Korn Temple in Khon Kaen Province has been developed through the collaboration of the temple, community, and villagers, which has aimed to strengthen river ecosystem management and improve fish farming along the river [41]. Some reserves created by separate communities in Thailand’s Salween Basin have markedly increased fish richness, density, and biomass relative to adjacent areas [42]. Freshwater Protected Areas in the Lower Mekong Basin have been established to meet a variety of objectives based on community needs and the goals of organizations and government agencies, including improving food security, alleviating poverty, and conserving biodiversity [40]. All these practices have strengthened the river ecosystem management, and promoted the sustainable development of freshwater basins. In terms of management objectives, these co-managed fish reserves mostly focus on fishery resources and aquatic biodiversity, whereas the more significant function of the Carp Brook ecosystem perhaps lies in its supply of cultural services formed by its rich cultural landscape. 

## 5. Conclusions

### 5.1. Chinese Traditional Views of Nature Is Important for Artificial Ecosystems

The concept of the view of nature refers to ideas and conceptions about how people treat nature and its resources, including various perspectives, attitudes, and ethical and moral conceptions [43]. The Carp Brook ecosystem is an artificial river ecosystem, and its construction and maintenance reflect the Zheng people’s view of nature. This view is formed by the integration of three aspects of traditional Chinese philosophy. Firstly, the purpose for the construction of the Carp Brook ecosystem reflects the concept of “Unity of Man and Nature”. The Zheng people realized that humans were a part of nature, and the relationship between human and nature was not to conquer and to be conquered, but to live in harmony. Hence, they bred carps but did not eat them, and built a harmonious relationship with carps for hundreds of years accordingly. Secondly, a series of ecological engineering measures to construct the Carp Brook ecosystem reflect the concept of “Conforms to the Nature”. The engineering measures were carried out based on the understanding and following the law of nature. They not only did not cause conflicts between human beings and nature, but also realized the long-term survival between humans and river ecosystems. At last, the measures for carp protection reflect the concept of “Love For Living Things”. The Zheng people believed that all living things, even animals, should be cherished. They sanctified the carps and protected them with strict rules. Furthermore, the carps were considered as humans in carp burials and sacrifice ceremonies. Under the guidance of these concepts, an artificial river ecosystem has been healthy for more than 800 years and has provided sustainable ecosystem services to human society. 

### 5.2. A Choice between Material and Immaterial Services Should Be Made Carefully

When making a choice between some different ecosystem services, people tend to prefer the material supply services that can bring economic value directly, which results in a contradiction between socioeconomic development and ecosystem protection [44,45]. Over more than 800 years, the Puyuan villagers have never fished and mined in the Carp Brook, but have constantly protected the river ecosystem and inherited the cultural elements along it. The Carp Brook ecosystem has provided regulating services and cultural services to mankind which have translated into human well-being effectively. With the development of society and economy, especially when most basic material needs of human beings are met, the demands for ecosystem immaterial services will continue to increase. It is necessary to choose more carefully between material supply services and immaterial services.

### 5.3. Traditional Folk Customs Have Strong Binding Forces in Ecosystem Protection

With a vast territory, there are significant differences between regions within China, especially in rural areas [46]. Accordingly, different measures are necessary to effectively protect the rural environment. Folk customs (e.g., village regulations) are aligned with local living habits and have a public opinion basis with strong regionalism and pertinence. The collective consciousness implied in folk customs plays an important role in restricting and regulating all aspects of rural life. Therefore, the traditional folk customs related to ecosystem protection should be fully explored and preserved. Furthermore, the improved version should serve as a useful supplement to the national environmental protection regulations.

## Figures and Tables

**Table 1:** Water quality indicators of the Carp Brook over 2020–2022.

	2020	2021	2022	Grade I Standard for Drinking Water Sources ^1^
Chroma	5.0000	<5.0000	10.0000	≤15.0000
Turbidity (NTU ^2^)	1.1000	0.4100	1.5400	≤3.0000
Foul smell	none	none	none	none
Volatile phenol (mg/L)	<0.0020	<0.0020	<0.0020	≤0.0020
Anionic synthetic detergent (mg/L)	<0.0500	<0.0500	<0.0500	≤0.3000
Sulfate (mg/L)	<0.7500	2.3320	0.8714	<250.0000
Nitrate (mg/L)	0.1822	1.2940	<0.1500	≤10.0000
Chloride (mg/L)	<0.1500	1.0190	0.8662	<250.0000
Fluoride (mg/L)	<0.1000	<0.1000	<0.1000	≤1.0000
Cyanide (mg/L)	0.0030	0.0090	0.0370	≤0.0500
Oxygen consumption (mg/L)	0.4800	0.8200	0.5800	≤3.0000
Ammonia–nitrogen (mg/L)	<0.020	0.4830	<0.020	≤0.5000
Total coliform group (MPN/100 mL)	0.0000	2.0000	0.0000	≤100.0000

^1^ Water Quality Standard for Drinking Water Sources (CJ 3020-93), approved by the Ministry of Ecology and Environment of the People’s Republic of China. ^2^ NTU: Nephelometric turbidity unit.

## Data Availability

No new data were created or analyzed in this study. Data sharing is not applicable to this article.
